# Stereology with OPEN-Stereo: low-cost, accessible, and accurate cellular quantification

**DOI:** 10.1038/s41598-025-33697-x

**Published:** 2025-12-30

**Authors:** Cassia Overk, William C. Mobley

**Affiliations:** Department of Neurosciences, University of California San Diego, La Jolla, CA 92093-0624 USA

**Keywords:** Stereology, Open source, Cell quantification, Machine learning, Coordinate mapping, Microscopy, Visual odometry, Biological techniques, Computational biology and bioinformatics, Neuroscience

## Abstract

Critical to rigor in neurodegeneration research is the accurate and unbiased assessment of degenerative phenotypes, where stereology remains the gold standard, yet its widespread adoption is hindered by the high cost of proprietary systems. We developed OPEN-Stereo, an open-source stereology platform that integrates standard microscopy hardware with intelligent, software-based calibration and positional control. Innovative use of computer vision methods, multi-scale image-based calibration and navigation, are combined with open-loop stage data to establish a global positioning system without costly hardware, while remaining within the accuracy tolerances inherent to stereological sampling. OPEN-Stereo implements the stereological random sampling method for unbiased cell counting. Validation using samples previously analyzed on commercial systems demonstrated up to 95% agreement in cell counts across multiple brain regions, with statistical equivalence confirmed by two-way repeated measures ANOVA (p = 0.8962). Beyond enabling accessibility, OPEN-Stereo’s image-analytic architecture enables stereology to evolve beyond manual practice, toward AI-driven cell identification and counting.

## Introduction

Stereology is a rigorous unbiased stochastic population sampling method^1,2^. At its most abstract level, stereology is a systematic approach to counting and quantifying particles within a three-dimensional volume by way of stochastic sampling of sub-volumes to obtain an unbiased estimate of the feature population within the larger volume. This provides a framework for estimating the characteristics of complex structures or particles, even when only a portion of the volume can be observed or measured. In essence, stereology provides access to quantitative measurements within a three-dimensional world of particles by carefully selecting and analyzing a fraction of the encompassing space. This abstraction is useful in various scientific fields, including biology, materials science, geology, and more, where it is often impractical to count every particle or cell individually. Stereology estimators often rely on assumptions about the distribution of particles within the volume^1^. This assumption is critical because stereological methods extrapolate from samples to estimate the properties of the larger volume. The population estimation process is designed to prevent overestimation or underestimation of the true number of particles.

Stereology has been applied across many subfields of biology and is essential for ensuring rigor and transparency in the quantitative evaluation of data in both model systems and human cases^3,4^. For example, the development and implementation of unbiased stereology has provided a rigorous method to quantify the number of cells in a particular region^5^, estimate changes in the change in the number of cells associated with neurodegeneration^6^, and has been widely implemented in fields beyond neuroscience^7^. The landmark publication under the pseudonym D.C. Sterio detailed the method on which current point count stereology applications are based^1^. Although this method can be implemented using photomicrographs, the modern application can be very costly, utilizing proprietary software that overlays a digital counting frame on a live image of the tissue sections visualized on a computer screen from a microscope and, more recently, virtual reconstruction of scanned slides. Addressing challenges in stereology would benefit from the availability of open-source tools and training.

There are several systems available for performing unbiased stereology techniques, as reviewed^3^. A widely adopted implementation of unbiased stereological cell number estimation is the optical fractionator^8^. Commercial stereology systems require proprietary software and specialized hardware equipment, including a system-specific camera, stage, and z-encoders. While these systems offer a wide range of stereological probes and workflows, the high cost of the system software may leave this product out of reach for users. While there is no open-source/free software that is fully integrated with the microscopy hardware to create a stereological system for researchers, there is a partial analysis component, that offers post-image-acquisition analysis functions for already acquired tissue section imaging data, for example STEPanizer^9^. Assembling a full stereological solution using a partial analysis component like STEPanizer requires extensive digitization of tissue. The cost of digitization and storage at the required scale for high-resolution images on a per experiment basis is also resource prohibitive and beyond practical reach for most users. Developing a low-cost stereology system that utilizes existing research microscopy hardware without the need to acquire specific high-cost hardware and/or software would address a major hurdle in the availability of quantitative, unbiased, and rigorous analysis of pathological changes.

Accordingly, we developed a scalable open-source stereological software system with reduced system requirements and at a fraction of the cost of commercial stereology systems. This system can run on standard microscopy hardware widely available in laboratories or available at a fraction of the original cost on resale sites such as Ebay. We acquired statistically equivalent stereological data using innovative analytical components and well-established computer vision methods^10–12^ like automatic feature tracking and multi-scale camera calibration that make use of camera images in combination with open-loop stage positioning data to replace the functionality of feedback-based 3D positioning sensors. An additional benefit to developing an open-source system architecture that decouples hardware from software protocols is that it more naturally allows integration of AI/machine learning based components to automate and speed up the manual stereology tasks like feature/cell detection and counting. In the following sections, we describe the implementation of a cell/point counting system using minimal precision microscopy elements integrated through intelligent software engineering. This approach combines the advantages of precision computer microscopy imaging and modern open-source computer vision tools with implementation of a systematic and unbiased method of stereology. The open-source stereology system is built on a few, but powerful elements. These include two calibration levels, linking XY-stage motor control to the camera, and ultimately the camera pixel spaces between different objectives. At the highest level of system hierarchy is the implementation of the stereology algorithm that guides the user through a semi-automatic cell counting process. To demonstrate reproducibility and statistical equivalence with currently available commercial systems, we analyzed the same tissue sections using our system compared with an existing commercial system. We detail herein a freely available stereology program, accompanied with video tutorials, that does not require specialized expensive hardware, but rather leverages standard microscope equipment.

## Results

### Overview of the implementation of image feature-based calibration and multi-objective image registration

Stereology requires the user to outline a region of interest (ROI) at a low magnification and then switch objectives to a higher magnification while maintaining the fidelity of the outlined ROI. Moreover, as the stage moves, the overlaid visual representation of the outlined regions on the screen needs to track with the movement of the tissue. Therefore, a global XYZ stage positioning system and multi-scale calibration strategy are needed to address this fundamental requirement of stereology. We utilized established computer vision techniques, specifically in the realms of feature tracking and visual odometry^10,11^, to implement calibration functions. These methods are frequently employed in the artificial vision subsystems of self-navigating robots. In our system, they are coupled with the motorized stage data to achieve robust and reproducible spatial registration across magnifications. While the stepper motors provide precise movement within the stage’s XY plane, users lack direct visual feedback for accurate navigation, feature selection, or ROI tracking, often relying on rough estimates or guesses when selecting or attempting to return to fiducial points or ROIs that lie outside the current field of view, as is typically the case when navigating large samples across scales. To bridge this gap, visual feature-based calibration establishes a spatial mapping between motor coordinates and the microscope camera’s image space. Because users interact with the sample entirely through the objective and live camera feed, this mapping anchors motor space to the user’s visual frame of reference. It enables intuitive and repeatable navigation to previously selected features and regions, via downstream ROI and feature-point selection mechanisms by placing precise, multi-objective spatial tracking under user control. The calibration step provides the foundational spatial logic that unlocks intuitive, repeatable navigation and underpins downstream stereological algorithms that rely on user-directed sampling and decision-making.

### Establishment of a global coordinate system

At the core of the system is a global coordinate system that serves as a unified XYZ reference frame for all geometric features and reference points, selected either by the user or the system based on object appearance in the camera image space. The 3D spatial components $(X_g, Y_g, Z_g)$ remain invariant, independent of the x-y motion control sensors (i.e. XY stage stepper motor positions) and the selected objective magnification. This ensures that every feature or point on the microscope slide or tissue space is uniquely defined within a single, stable coordinate system. Consequently, user-defined points can be accurately accessed and revisited, regardless of magnification changes or x-y stage positioning, whether adjusted automatically or manually via the joystick.

### Mapping pixel coordinates to global coordinates

The calibration function of (Eq. 1) within the OPEN-Stereo code, maps the actual pixel coordinates, $\textbf{XY}_{\text {pixel}} = [X_{\text {pixel}}, Y_{\text {pixel}}]^T$, on the camera image pixel space to the global coordinates, $\textbf{XY}_{\text {global}} = [X_g, Y_g]^T$, of what is currently visible on that pixel, while accounting for the objective magnification, or zoom level, (through an integer $\text {Zoom}_{\text {index}}$; Supplemental Fig. 1).

1 $$\begin{aligned} \textbf{XY}_{\text {global}} = \textbf{F}_{\text {global}}(\textbf{XY}_{\text {motor}}, \textbf{XY}_{\text {pixel}}, \text {Zoom}_{\text {index}}) \end{aligned}$$

This allows the user to identify the exact global position of points selected on the screen as part of geometric features, such as a cell nucleus, or sets of points defining region boundaries within the tissue on the slide.

### Mapping global coordinates to XY stage movements

The conversion function mapping the position of the stage, the screen pixel location, and the objective magnification on to a global coordinate (Eq. (2)) is critical for precision and automatic positioning and navigation within the space of user-selected stereological point features (Supplemental Fig. 2). Given the global coordinates, $\textbf{XY}_\text {global}$, of a specific point of interest (e.g., the location of a cell), the function calculates the necessary x-y stage movements to position the target feature point at the center of the camera’s field of view, specifically at the midpoint coordinates (800, 512) for a camera resolution of $1024 \times 1600$, in our particular case. This feature ensures that any user-defined point can be revisited with precision, making it possible to return to previously selected locations on the microscope slide or tissue space accurately. The ability to revisit specific points with precision is crucial in stereology.

2 $$\begin{aligned} \textbf{XY}_{\text {motor}} = \textbf{F}_{\text {motor}}(\textbf{XY}_{\text {global}}, \textbf{XY}_{\text {pixel}}, \text {Zoom}_{\text {index}}) \end{aligned}$$

The system’s accuracy is significantly enhanced by the use of high-fidelity calibration, combined with modern, high-precision and high-quality XY-stagers. This is critical to ensuring the reliability of stereological analyses.

### Calibration architecture

The camera calibration process helps to establish the global coordinate system and ensures the accuracy and reproducibility of measurements, thus providing the foundation for the implementation of the stereology algorithm. Calibration between different objectives in microscopy involves establishing a precise geometric transformation between the images obtained at various magnifications. This ensures that scale and positional information remain consistent when switching between objectives and changing the zoom level of the microscope.

Providing a simple and fully formalized computational calibration step is critical to our system. Calibration across multiple objectives satisfies the geometric requirements for the transformational mapping function for implementing stereology algorithms. By accurately mapping pixel coordinates between objectives, the system ensures that features identified at low magnification (e.g., 4× for structural annotation) can be revisited with high magnification (e.g., 100× for cell counting) without loss of spatial correspondence. This capability demonstrates the establishment of a unified, objective-invariant coordinate system that supports seamless multi-scale navigation and analysis. This alignment allows straightforward programming and implementation of the geometric transformation algorithms, as points maintain their spatial integrity across different objective magnifications. This eliminates the need for manual scale adjustments, streamlining the stereological process. The two fundamentally different, and core calibration programs in the system are described below.

### XY calibration within the same scale (objective) and calculation of calibration matrix $\textbf{K}(z_k)$

The first level of calibration establishes the spatial relationship between the camera’s XY pixel space and the XY stage motor coordinate system. It computes how pixel displacements observed in the image correspond to physical displacements commanded through the motorized stage. This mapping enables accurate stage movements based on user-selected image features and underlies key system functionality, such as centering the focal plane on a target feature identified by its global coordinates. It also supports manual point selection directly from the live camera view, by combining clicked pixel coordinates with the current stage position and zoom level to compute the corresponding global spatial coordinates. The mathematical formulation of this image-to-stage transformation, including the underlying computer vision algorithms, is described in the following sections.

The brightness constancy equation (Eq. 3) underlies feature tracking between consecutive image frames acquired at a fixed objective, and is central to our calibration pipeline within the same magnification scale. It assumes that the brightness (or intensity) of a feature remains unchanged across frames, provided illumination and material properties are consistent. This principle is leveraged by optical flow methods such as the Lucas–Kanade algorithm^13^, which enables robust estimation of image displacement due to stage motion. Mathematically, the brightness constancy equation is expressed as:

3 $$\begin{aligned} I(x, y, t) = I(x + \Delta x, y + \Delta y, t + \Delta t). \end{aligned}$$

Here, *I*(*x*, *y*, *t*) represents the intensity *I* of a pixel at position (*x*, *y*) at time *t*. $I(x + \Delta x, y + \Delta y, t + \Delta t)$ represents the intensity of the corresponding pixel at position $(x + \Delta x, y + \Delta y)$ at time $t + \Delta t$.

**Fig. 1:**
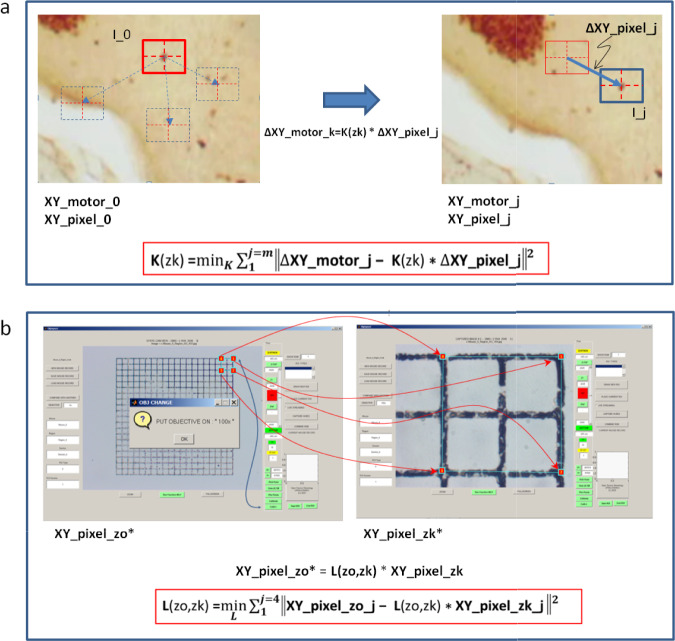
Calibration and feature tracking at a fixed magnification and between magnifications. (a) Calibration for $\mathbf {K(zk)}$. The calibration pipeline using a series of images automatically collected from a predefined stepper motor movement command. The user selects one unique trackable feature point (must be a corner/edge) on the image ($I_0$) using a 10× objective. The feature point must fit inside the tracking window represented as the solid outlined red or blue square. The stepper motor moves the image to a predefined location. Using the brightness constancy equation, the same feature is visually identified by its unique pixel intensity. This process repeats at least 4 times for the feature point (the user can increase the repetitions for increased accuracy). The pixel location and the stepper motor coordinates are then associated in a matrix $\textbf{K}$ at the specified magnification. (b) Calibration for $\mathbf {L(zo,zk)}$. Calibration and feature tracking between magnifications. The $\textbf{L}$ matrix is calculated by selecting four points on a calibration grid ($\textbf{XY}_{\text {pixel}, z_o, j}$, shown as red squares) at the first magnification (10×; left panel), and then reselecting those same points in the same order at the second magnification (100×; right panel; points respectively identified by red arrows). $\mathbf {L(zo,zk)}$ is a three by three calibration transformation matrix between reference objective $\textbf{zo}$ and the objective level $z_k$, such that $\textbf{XY}_{\text {pixel}, z_o} = \textbf{L}(z_o, z_k) \cdot \textbf{XY}_{\text {pixel}, z_k}$. The $\textbf{L}$ matrix maps the changes in pixel locations between two magnifications. This establishes the pixel coordinate mapping between two magnifications.

The feature tracking algorithms (Eq. 4,^10^) leverage this equation to estimate the motion or displacement $(\Delta x, \Delta y)$ of image features between two consecutive frames by analyzing changes in pixel intensity values. Feature tracking algorithms search for corresponding pixels in the two frames to establish feature correspondences and calculate the motion vectors. This principle is used to create the calibration pipelines. Once we are able to calculate the displacement $(\Delta x, \Delta y)$, denoted with $\Delta \textbf{XY}_{\text {pixel},k}$ (Fig. 1a), we can use a series of auto-generated image-based displacements to calibrate the relationship between pixel coordinates and the XY stage stepper motor coordinate system using the optimization equation below:

4 $$\begin{aligned} \textbf{K}(z_k) = \arg \min _{\textbf{K}} \sum _{j=1}^m \left\| \Delta \textbf{XY}_{\text {motor},j} - \textbf{K}(z_k) \cdot \Delta \textbf{XY}_{\text {pixel},j} \right\| ^2 \end{aligned}$$

Here $\Vert \cdot \Vert$ denotes the Euclidean norm (i.e., vector length), and the squared norm represents the sum of squared differences across both dimensions. This is a least-squares error minimization problem that is solved for the calibration matrix $\textbf{K}(z_k)$ using the pseudo-inverse solution functions natively supported in MATLAB^14^. The resulting matrix $\textbf{K}(z_k)$ represents a 2D affine transformation that captures rotation, scaling, and shear between the image pixel space and the XY stage motor coordinate space at a given magnification.

### Mapping between pixel coordinates of different objective scales and calculation of inter-scale calibration matrix $\textbf{L}(z_o, z_k)$

The second level of calibration (Eq. 5) establishes the geometric transformation between pixel coordinate systems at different objective magnifications. For example, a user may identify a cell using a 4× objective and later revisit it using a 100×. This calibration computes the motor adjustments needed to center the same feature in the new field of view, enabling consistent spatial alignment across scales. It ensures that the view in the 100× objective scale centers on the same cell or feature initially identified using the 4× objective scale.

5 $$\begin{aligned} \textbf{L}(z_o, z_k) = \arg \min _{\textbf{L}} \sum _{j=1}^4 \left\| \textbf{XY}_{\text {pixel},z_o,j} - \textbf{L}(z_o, z_k) \cdot \textbf{XY}_{\text {pixel},z_k,j} \right\| ^2 \end{aligned}$$

Because brightness and contrast vary across objectives, intensity-based tracking can become unreliable. Instead, we use user-defined point correspondences to compute the affine transformation between objective scales. To account for translation, pixel coordinates are represented in homogeneous form $[x, y, 1]^T$, and $\textbf{L}(z_o, z_k)$ is defined as a $3 \times 3$ affine transformation matrix^11^. A minimum of four non-collinear calibration points is required to solve for the eight degrees of freedom in this 2D transformation. These points are used in a least-squares fit (Fig. 1b), analogous to the method used to compute $\textbf{K}$ in Eq. (4). Once estimated for the specific camera and stage setup, $\textbf{L}(z_o, z_k)$ enables the projection of user-selected pixel coordinates between objective scales, ensuring consistent spatial alignment across magnifications. Because all objective magnifications are registered to a single reference objective $z_o$, only $n$ calibrations, between each $z_k$ and $z_o$, are needed. This avoids the need for calibrating every possible pair of objectives, reducing the number of required transformations from all possible objective pairs to just one per objective. Please note: Greenough stereo microscopes and poor camera reducers violate local affinity, and therefore should not be used.

### Transformations for intra- and inter-objective calibration and the global coordinate system

The intra- and inter-objective calibration algorithms described above are implemented to extract affine transformation matrices that define the mapping between image pixel coordinates and physical stage coordinates. The first calibration level (Fig. 1a) establishes precise control and alignment within the same magnification scale, enabling both automated accuracy and user-guided flexibility in point selection. The second level (Fig. 1b) ensures spatial consistency across different objectives, allowing users or the system to revisit the same point of interest at varying magnifications with precision.

**Fig. 2:**
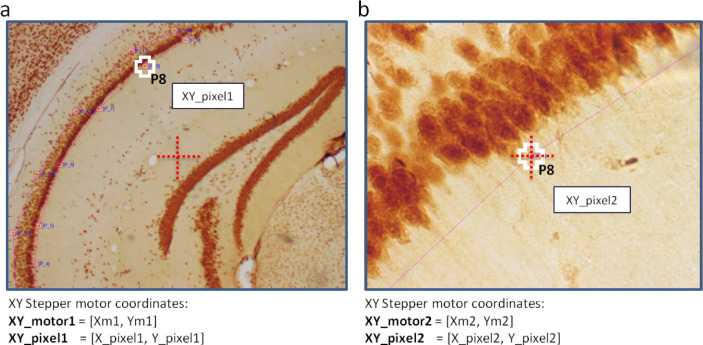
Mapping between screen pixel coordinates and global motor coordinates. Navigation across magnifications is enabled by combining the functions $\textbf{F}_{\text {global}}$ and $\textbf{F}_{\text {motor}}$. (a) A user selects a point of interest at 10× magnification ($P_8$, marked by the white cross), and (b) revisits the same feature at 40×, with the feature now centered in the field of view (red dashed cross). When a user clicks on the screen, the function $\textbf{F}_{\text {global}}(\textbf{XY}_{\text {motor},1}, \textbf{XY}_{\text {pixel},1}, \text {Obj} = 10\times )$ converts the screen pixel coordinate into a global motor position $\textbf{XY}_{\text {global}, P8}$. To bring that feature back into view at a different objective, the system uses $\textbf{F}_{\text {motor}}(\textbf{XY}_{\text {global}, P8}, \textbf{XY}_{\text {pixel}}, \text {Obj} = 40\times )$ to solve for the new motor position that maps the known global point to a desired location on screen. For convenience, the system uses the center of the field of view as the screen target, setting $\textbf{XY}_{\text {pixel}} = [512, 512]$. This process relies on the transformation defined in Eq. (6), which incorporates a pixel-to-motor scaling matrix $\textbf{K}(z_0)$ and an affine mapping $\textbf{L}(z_0, z_k)$ to maintain spatial consistency across objectives.

Together, these calibration levels give rise to a global coordinate system and an objective-invariant position vector $\textbf{XY}_{\text {global}}$ that maps screen-selected points to a shared spatial reference frame. This global coordinate is a function of the current XY stage position $\textbf{XY}_{\text {motor}}$, the calibration matrix for the reference magnification $\textbf{K}(z_o)$, and the inter-objective transformation $\textbf{L}(z_o, z_k)$, where $z_k$ is the objective used to select the feature point, and $\textbf{XY}_{\text {pixel}, z_k, \text {user}}$ is the clicked screen coordinate (Eq. 6; Fig. 2) ($\textrm{proj}_{2D}$ denotes projection from homogeneous coordinates $[x, y, 1]^T$ to 2D Euclidean space $[x, y]^T$ by removing the third dimension. In Eq. (6), this operator appears after $\textbf{L}$ to maintain mathematical correctness, as $\textbf{K}$ is defined only over 2D coordinates. While implementation-specific pipelines may optimize or collapse this step, the formulation here prioritizes conceptual clarity and consistency across coordinate spaces.):

6 $$\begin{aligned} \textbf{XY}_{\text {global}} = \textbf{XY}_{\text {motor}} + \textbf{K}(z_o) \cdot \textrm{proj}_{2D}^1 \left\{ \textbf{L}(z_o, z_k) \cdot \textbf{XY}_{\text {pixel}, z_k} \right\} \end{aligned}$$

### OPEN-Stereo workflow and user interface

This section describes the implementation of the OPEN-Stereo workflow and stereological algorithm within it. The algorithm leverages a simplified global coordinate system to enable accurate quantification of cells within a 3D volume and supports intuitive user interaction throughout the counting workflow.

#### Graphical user interface

We implemented the GUI in MATLAB to provide streamlined access to core computer vision algorithms and to control the imaging hardware, including the camera and motorized stage. MATLAB enables low-level serial communication with the XY and Z stages and supports HTTP-based access to USB cameras, facilitating integration with commonly available hardware. This dual-mode communication eliminates the need for proprietary SDKs and allows flexible system configuration. A walkthrough video is available on GitHub (“Open-Stereo GUI walkthrough”) for further reference ^15^.

#### Calibration prerequisites

Before proceeding, the system must be calibrated to map pixel coordinates to stage coordinates. This includes estimation of the intra-objective matrix $\textbf{K}(z_k)$ and inter-objective transformation $\textbf{L}(z_o, z_k)$ as described above. The workflow that follows assumes these calibrations are complete.

#### Defining the region of interest

**Fig. 3:**
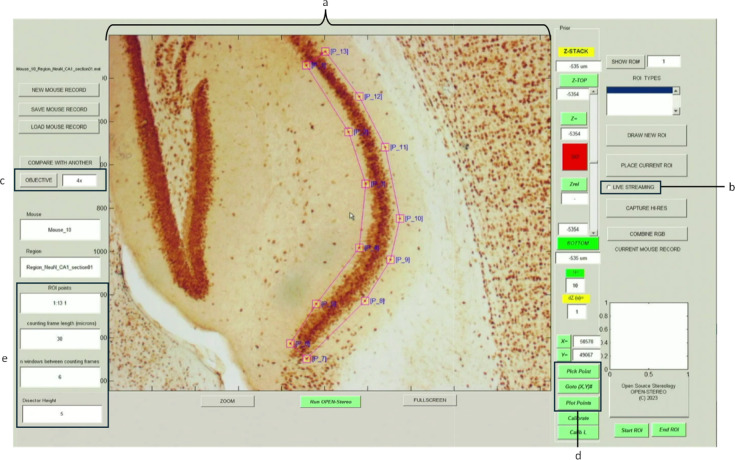
Screenshot of the OPEN-Stereo GUI showing the CA1 brain region outlined at low magnification. All components of the GUI along with an initial outline, using a hippocampal CA1 brain region as an example. Components of the GUI include: (**a**) live view image, (**b**) live streaming (on/off), (**c**) magnification selection, (**d**) ”Pick Point” button, ”Go To XY#”, and and ”Plot Points” button, (**e**) user input areas for the ROI points, length of the disector (in µm), and number of disectors between sampling sites.

The interface displays a live image of the specimen (Fig. 3a), and the [Live Streaming] button (Fig. 3b) toggles real-time acquisition. After selecting a low-magnification objective (e.g., 4×; Fig. 3c), users define a region of interest (ROI) by selecting reference points using the [Pick Point] tool (Fig. 3d). These clicks are registered on the screen display and rendered using the [Plot Points] function to visually outline the ROI.

#### Configuring stereological sampling parameters

After ROI selection, the user switches to a higher-magnification objective (typically 100×) for detailed cell counting. The interface allows configuration of key sampling parameters: the global window size (in microns), selection of ROI points, disector size (via “Counting Frame Length”), spacing between frames (as a multiplier of frame length), and the disector height (Fig. 3e). These parameters define the 3D sampling volume and ensure reproducibility across sessions and users.

#### Stereological sampling and cell counting

The core of our stereological algorithm is the systematic sampling of the ROI. Building on the principles of DC Sterio^1^, the algorithm generates a grid of sampling points within the user-defined region. These grid points are distributed uniformly throughout the 3D volume corresponding to each XY square grid cross-section, ensuring representative and unbiased sampling^16^.

**Fig. 4:**
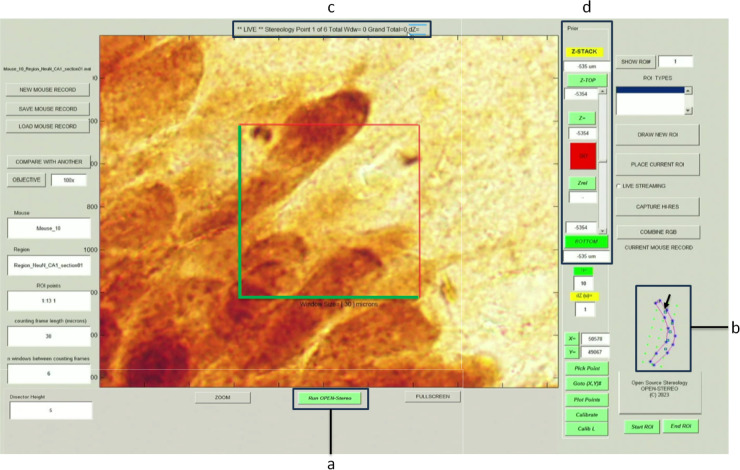
Screenshot of the OPEN-Stereo GUI running the stereology counting program. Once the ROI is defined, and the user has selected the high magnification, (**a**) pressing the “Run OPEN-Stereo” button starts the stereology point counting program. The live screen updates in the GUI along with a complete outline of the hippocampal CA1 region on the right-hand side. (**b**) The current location is marked on the outline along with all counting locations. (**c**) Information is also provided at the top of the GUI, including that the image is “LIVE,” the current counting window, the number of points counted, the current site, and the total number of points counted. (**d**) Z-positioning panel for identifying the ”top” and ”bottom” of the tissue section, current z position, and the measured section thickness ”z-rel”.

The user is guided through each grid point by running the program (Fig. 4a). At each grid point, the user is prompted to perform cell counting within a specified 3D volume. This stepwise approach ensures the systematic and unbiased quantification of cells within the ROI, leading to statistically valid results. The GUI displays a complete model outline of the ROI (Fig. 4b), the different sampling sites (Fig. 4b, arrow: blue squares). At the top of the program (Fig. 4c) is the current state of the system (“Live”), the current site location out of the total number of sites (”Stereology Point 1 of 6”), the current number of markers at the current site (”Total Wdw = 0”), the total number of markers counted for the section (”Grand Total = 0”), and the measured tissue thickness (”dZ = ”).

The user then uses the Prior panel (Fig. 4d) to focus on the top of the focal plane and press the [Z-TOP] button (Fig. 4d), and then the user changes the focal plane to the bottom focal plane and presses the [Bottom] button (Fig. 4d). The thickness of the sampling site is displayed under the [Zrel] button (Fig. 4d). To confirm that the correct focal planes were assigned to top and bottom, the [GO] button will turn green from red (Fig. 4d). Additionally, based on the user-defined height of the counting frame, the [GO] button will turn red when above or below the upper or lower bound of the sampling grid, respectively (Fig. 4d).

The user can place markers on the cells by pressing the left mouse button. After completing the current sampling site, middle-clicking the mouse will automatically move the stage to the next sampling site, and the user continues counting. Right-clicking will remove the most recent marker and adjust the marker count. When all of the sampling sites are completed, the system will display the total number of markers at the top (Fig. 4c).

### Validation of OPEN-Stereo

A core principle of unbiased stereology is that analyzing the same tissue, despite reasonable differences in counting parameters, should yield comparable estimates of total cell number. Because stereology quantifies biological counts rather than spatial coordinates, we used count reproducibility as the primary validation endpoint, since it inherently integrates all spatial, optical, mechanical, and user-derived sources of variation. Ultimately, these total cell estimates are the output that stereology is designed to produce, making their reproducibility the most meaningful measure of system performance. Therefore, to validate OPEN-Stereo, we used a mixture of 12 mice that were part of different experiments. To avoid ambiguity, we note that the use of the term ‘validation’ refers solely to empirical evaluation of system accuracy and stability for research applications, distinct from the formal regulatory meaning of validation under 21 CFR Part 11. Cases 1-8 were from experiments evaluating the number of NeuN positive cells in mouse hippocampal CA1 and CA3 brain regions, while cases 9-12 (Table 1) are a subset of previously published cases^17^. All cases were re-evaluated blind to the previous counts. Moreover, for additional rigor, some cases (cases 5-12) were evaluated using different counting frame and sampling grid sizes between the two stereology systems. We found a high level of congruence between the estimated number of cells using the OPEN-Stereo system compared to a commercial system. In the hippocampal regions of the CA1 and CA3, the median congruence was 95% and 88%, respectively; and 83% in the medial septum (MS) (Table 1). A two-way repeated measures ANOVA analysis comparing the data from different brain regions and methods indicated that the two methods were statistically indistinguishable ($p = 0.8962$).

**Table 1:** .

Case number	Antibody	Brain region	Every nth section	Counting frame		Sampling grid area		Raw count		Estimated number of cells		% Congruence
				Commercial	Open-Stereo	Commercial	Open-Stereo	Commercial	Open-Stereo	Commercial	Open-Stereo	
1	NeuN	CA1	12	30 × 30	30 × 30	150 × 150	150 × 150; k=5	159	153	86127	82620	95.8
2	NeuN	CA1	12	30 × 30	30 × 30	150 × 150	150 × 150; k=5	132	137	74551	77268	96.4
3	NeuN	CA1	12	30 × 30	30 × 30	150 × 150	150 × 150; k=5	149	159	84699	90830	93.2
4	NeuN	CA1	12	30 × 30	30 × 30	150 × 150	150 × 150; k=5	164	136	92349	76704	81.5
5	NeuN	CA3	12	83 × 83	30 × 30	200 × 200	180 × 180; k=6	107	116	92513	95213	97.1
6	NeuN	CA3	12	83 × 83	30 × 30	200 × 200	180 × 180; k=6	498	106	72218	93416	74.4
7	NeuN	CA3	12	83 × 83	30 × 30	200 × 200	180 × 180; k=6	421	70	60168	62294	96.5
8	NeuN	CA3	12	50 × 50	30 × 30	200 × 200	180 × 180; k=6	130	97	106579	87160	80
9	ChAT	MS	6	85 × 85	40 × 40	140 × 140	80 × 80; k=2	102	69	2526	2070	80.2
10	ChAT	MS	6	85 × 85	40 × 40	140 × 140	80 × 80; k=2	80	79	2073	2370	86.6
11	ChAT	MS	6	85 × 85	40 × 40	140 × 140	80 × 80; k=2	149	67	3529	2665	72.1
12	ChAT	MS	6	85 × 85	40 × 40	140 × 140	80 × 80; k=2	103	70	2319	2100	90.1

## Discussion

Accurate and unbiased quantification of neuronal populations is crucial for rigorous investigation of neurodegenerative disorders. Stereological assessment remains the gold standard for this purpose, but access has been limited due to the high costs and technical demands associated with commercial stereology systems. In this sense, alternatives to overcome this problem should be considered.

Our novel open-source stereology software system overcomes these barriers, making quantitative neuroanatomical analysis more accessible to a broader range of researchers who may not otherwise be able to perform stereology-based experiments. The implementation of our stereological algorithm employs a simplified global coordinate system, credited to the calibration paradigm and choice of global positioning coordinate system described earlier to automate and simplify the user effort and streamline the cell counting process. Users can accurately quantify cells within a 3D volume by following a systematic procedure that encompasses region identification, parameter specification, and a combination of computer-guided automated volume sampling and manual cell counting sequence based on well-established stereological principles. This implementation contributes to the rigorous and unbiased analysis of complex biological structures within the specified ROI. Beyond neuroscience, our open-source stereology solution has potential applications in diverse fields requiring quantitative analysis of microscopic structures, such as materials science, biology, and pathology. The modular nature of our open-source software architecture allows for adaptability and customization, enabling researchers to tailor the system to their specific needs and imaging modalities.

A key strength of our approach is its cost-effectiveness and scalability. By leveraging standard Windows computing systems and widely available microscopy hardware, we have eliminated the need for expensive specialized equipment required by commercial solutions. In fact, the most expensive components can easily be acquired from laboratory equipment surplus stores, academic storage, or eBay. Operating at a fraction of the cost, our system enables laboratories with limited resources to conduct stereological studies that were previously unfeasible due to budgetary constraints. Furthermore, the ability to run multiple instances simultaneously on inexpensive hardware facilitates larger-scale studies and higher throughput. While these are all strengths, they are at the same time, limitations in that the components are older, may not be supported by the manufactures, and the software runs using Matlab 2010a. Future iterations of OPEN-Stereo may be upgraded to Python and run on more recent computer hardware.

One noticeable difference is that the user never needs to create a reference point before tracing the ROI. This is made possible by the global coordinate system, which eliminates the need to manually align ROIs to fiducial landmarks on the slide. Importantly, this high-fidelity calibration framework supports repeatable and unbiased return to any spatial location, regardless of magnification or acquisition session. As such, it enables blinded reanalysis, multi-rater reliability, and validation experiments without the need for custom alignment procedures, features that are critical for rigorous quantitative biological analysis.

Central to our system design is the innovative integration of analytical components and computer vision techniques. By harnessing the information directly from images in combination with open-loop stage positioning data, we have effectively replaced costly high-precision 3D positioning sensors. One notable difference is that the user never needs to create a reference point before tracing the ROI. This small feature, due to the global coordinate system, means that the user will not have to align the ROIs to the slide. Equally important, images, sampling sites, and cell annotations are stored and accessed through explicit, transparent data structures in the code, so that the full image and coordinate workflow is visible and modifiable to the user. This integration of smart software with standard hardware components exemplifies the power of developing cost-effective solutions to overcome instrumentation limitations. Our validation comparing previously analyzed samples using a commercial system further underscores the reproducibility and statistical equivalence of our approach.

Looking ahead, the open image-analytical architecture of our system creates the opportunity for incorporating AI and machine learning capabilities and general crowd-sourced evolution of the system further into the future. Although the current implementation is fully manual with respect to cell detection and counting, the way images, masks, and coordinate tables are exposed in the software is deliberately “AI-ready”: external algorithms can directly access the same image data and annotation structures used for stereological sampling. Automated cell detection and counting algorithms could significantly enhance the efficiency and reproducibility of stereological workflows. Machine learning models trained on large datasets of annotated neuronal images using tools such as Fiji^18^ could potentially automate labor intensive manual processes, reducing human error and increasing throughput. Because the codebase is open and the data flows from image acquisition to annotation are explicit, we specifically invite interested users to inspect, reuse, and extend these components when developing such AI-based modules. Furthermore, such AI-driven components could be continually refined and updated, keeping our system at the cutting edge of technological advancements.

In conclusion, our open-source stereology software system addresses long-standing accessibility and cost barriers that have limited the widespread adoption of this critical quantitative methodology. OPEN-Stereo does not seek to replace commercially available systems, but offers a low-cost alternative for laboratories that cannot access them. Its primary impact is enabling stereology where it would otherwise be infeasible. By leveraging innovative analytical techniques, validating results against commercial systems, and providing an architecture that is transparent and extensible enough to support future AI/machine learning integration, our solution represents a significant step toward expanding access to rigorous neuroanatomical analysis tools. As we continue to refine and enhance our system, we envision a future where accurate and unbiased quantification becomes increasingly attainable, driving deeper insights into the mechanisms underlying neurodegenerative disorders and informing the development of effective therapeutic interventions. Notably, the development of this system during the resource-limited pandemic environment allowed researchers to maintain continuity in their work through inexpensively sourced, readily available hardware components such as older versions of Windows and MATLAB, as well as low-cost XY-stages and microscopy equipment. This resourcefulness, born out of necessity, has fueled innovation, demonstrating that constraints can be a powerful catalyst for developing accessible and impactful research tools.

## Materials and methods

### Microscope

A standard lab microscope capable of supporting an automatic stage and a camera mount is required. We chose an Olympus BX50 microscope (acquired on eBay). A set of objectives which at a minimum includes a low magnification of 4× to 10× and a high magnification of 60 to 100× with a high numerical aperture (N.A. 1.3-1.4). A full set of objectives came with the microscope and an Olympus 100× oil immersion objective (N.A. 1.3) was purchased separately from eBay.

### Automatic stage and controller

To ensure robust and reliable stage control, we chose the Prior Scientific XYZ stage system, known for its high quality and longevity. The serial command interface provided by the manufacturer is consistent across multiple ProScan models. We acquired a Prior ProScan I stage, controller, and joystick as pre-owned system components, and developed a complete software solution to interface with this hardware. Our implementation supports the Prior Scientific ProScan (I and II) XYZ stage systems. High-level graphical interface elements were created to abstract these commands, including XYZ actuators, position query handles, and automated motion routines to fiducial landmarks. At each marked location, X and Y positions and the current Z focus position are read from the Prior stage controller, and the Z coordinates are saved together with the X and Y coordinates. A ProScan stage is required for OPEN-Stereo due to the manufacturer defined serial motion command sets encoded in the software.

### CCD camera

Our goal was to make our stereology program as widely accessible as possible. We utilized a universal camera interface for accessing a wide variety of microscopy CCD/cameras from our open-source stereology software program. This was achieved by converting the typically USB-based CCD cameras into a web socket interface device through an open-source web-cam interface. We used the well-established YAWCAM system^19^. While any USB-based CCD camera will work with our software, we recommend investing in the highest quality camera that can be afforded.

### Computer system

The prototype was developed on an underutilized lab computer (Dell T3600, Windows 7) using MATLAB 2010a as the development environment. This demonstrated that the system software can be effectively implemented on older hardware, supporting the cost-saving claims. If required the MATLAB-based code is portable to newer versions and adaptable to other programming languages, ensuring long-term flexibility and maintainability. This proof-of-concept confirms that robust hardware–software integration is achievable using less expensive pre-owned systems without sacrificing functionality.

### Image acquisition

Image acquisition in the system is achieved using a USB-connected CCD camera, the open-source YAWCAM software, and MATLAB as the high-level interface. The architecture is organized as follows:

The microscopy setup includes a CCD camera, selected for its high sensitivity and image quality, characteristics well-suited to microscopy applications. The camera connects to the computer via a USB interface, offering a simple and reliable setup for image capture. YAWCAM, a free and open-source webcam software for Windows, serves as the control interface for the USB camera. It allows users to adjust camera settings, capture images, and stream video through a user-friendly interface. In our system, YAWCAM is configured to stream images over HTTP, effectively turning the camera into a live image server accessible via an internal web portal.

MATLAB is used to access and process these streamed images. Through standard HTTP requests, MATLAB retrieves image frames from the URL provided by YAWCAM. Once acquired, images are processed within the OPEN-Stereo interface using MATLAB’s built-in image processing functions. These images are then stored in a format suitable for downstream tasks such as camera calibration and stereological analysis. In summary, the image acquisition pipeline consists of a CCD camera streaming over HTTP via YAWCAM, and MATLAB retrieving and processing the images for use within the stereological system. This architecture enables robust, cost-effective, and flexible image handling for high-level stereological functionality.

### Integration of hardware and software

**Fig. 5:**
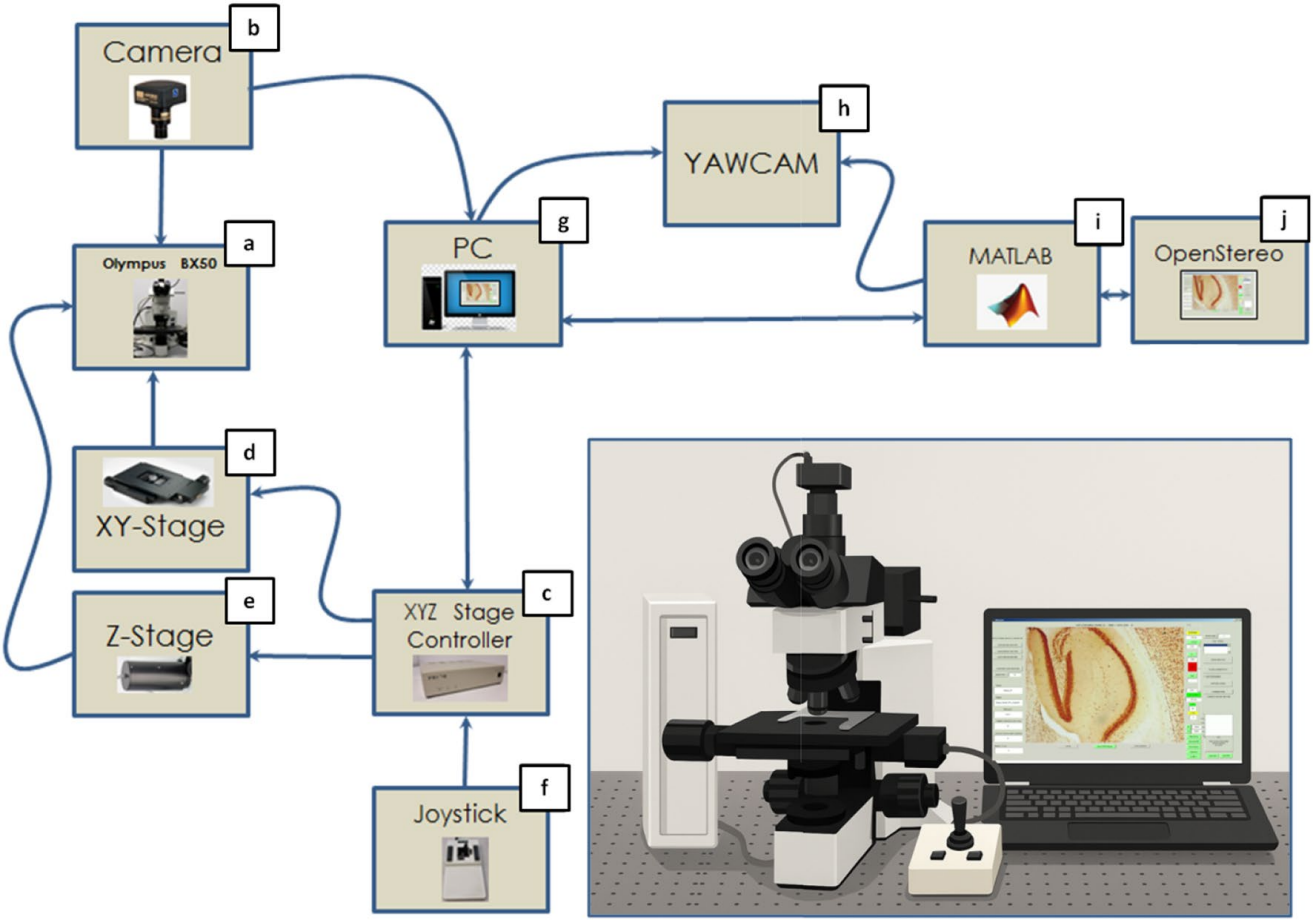
Component integration. The (**a**) microscope is connected directly to the (**b**) camera, (**c**) XYZ stage controller, (**d**) XY stage, (**e**) Z motorized stage, and (**f**) joystick. The (**b**) camera and (**c**) XYZ stage controller connect to the PC (**g**). The software running on top of the computer (**g**) includes (**h**) YAWCAM, (**i**) MATLAB, and (**j**) OPEN-Stereo.

The OPEN-Stereo system (Fig. 5) is centered around a computer (Fig. 5g) that orchestrates all hardware and software operations. It maintains bidirectional communication with both the MATLAB environment (Fig. 5i) and the XYZ stage controller (Fig. 5c) via serial interfaces, enabling seamless command and data flow. At the core of the optical system is an Olympus microscope (Fig. 5a), mounted with a USB-based CCD camera (Fig. 5b) and interfaced with motorized XY (Fig. 5d) and Z (Fig. 5e) stages. These stages provide precise movement across the slide and axial focus control. The XYZ stage controller (Fig. 5c) translates computer-generated commands into electrical signals to drive the stages and also connects to a manual joystick (Fig. 5f) for user-guided positioning when needed.

To facilitate image acquisition, the CCD camera streams data to the computer (Fig. 5g) via a USB interface. For broad compatibility and simplified control, the system uses YAWCAM (Fig. 5h), an open-source webcam software that converts the camera’s USB output into an HTTP stream accessible by MATLAB (Fig. 5i). MATLAB serves as the system’s software backbone, providing tools for image processing, data analysis, and hardware control.

The OPEN-Stereo application (Fig. 5j), developed within MATLAB, encapsulates the core stereology algorithms and user interface. It processes image streams from YAWCAM, communicates with the XYZ stage controller for navigation and focus, and supports real-time image acquisition and analysis. This tightly integrated system, including the microscope (Fig. 5a), camera (Fig. 5b), motorized stages (Fig. 5d,e), joystick (Fig. 5f), YAWCAM (Fig. 5h), MATLAB (Fig. 5i), and the OPEN-Stereo application (Fig. 5j), is coordinated through the central computer (Fig. 5g), providing a cohesive and user-friendly stereological quantification workflow.

### Stereology software system implementation

The stereology algorithm in the OPEN-Stereo system is designed to leverage the global positioning capability of the microscope stage, which allows the system to address and reliably repeat visits to any point on a microscope slide. This positioning capability is essential for implementing the systematic random sampling approach required for unbiased stereological quantification.

The stereology workflow begins with the user defining the region of interest (ROI) on the microscope slide, typically a brain region or tissue section. The system then generates a series of sampling locations within the ROI using a systematic random sampling scheme^20^. This scheme ensures that the sampling locations are distributed uniformly and randomly throughout the ROI, minimizing the potential for bias.

At each sampling location, the system employs a disector probe, which is a three-dimensional counting volume defined by the area of the counting frame and the height of the optical disector. The disector probe is positioned at the sampling location using the global positioning capability of the stage, ensuring precise and repeatable positioning (Supplementary Fig. 1). Within the disector probe, the user uses the fine Z-motor focus to evaluate the live image with the disector probe overlayed. Using the principles of stereology, the user counts cells that meet the counting criteria.

The cell counts obtained from each disector probe are recorded and used to calculate the numerical density of cells within the ROI (Eq. 7). This calculation considers the sampling fractions associated with the systematic random sampling scheme, including the section sampling fraction (ssf), the area sampling fraction (asf), and the height sampling fraction (hsf).

7 $$\begin{aligned} N = \sum Q \cdot \frac{1}{\text {ssf}} \cdot \frac{1}{\text {asf}} \cdot \frac{1}{\text {hsf}} \end{aligned}$$

where $N$ is the estimated total number of cells, and $\sum Q$ is the total number of cells counted within all sampled fields of view (disectors).

By leveraging the global positioning capability of the microscope stage, the OPEN-Stereo system can efficiently and accurately navigate to the predetermined sampling locations, ensuring that the disector probes are positioned precisely and consistently throughout the ROI. This approach minimizes the potential for bias and improves the accuracy and reproducibility of stereological quantification.

Overall, the implementation of the stereology algorithm in the OPEN-Stereo system combines the advantages of precise global positioning and systematic random sampling to provide an accessible and efficient solution for unbiased quantification of microscopic structures, such as neuronal populations in brain tissue.

### Validation experiments for OPEN-Stereo

We validated OPEN-Stereo using mouse brain sections that were previously assessed in the medial septum (MS) using a commercial stereology counting system^17^. A detailed description of the acquisition and preparation of the brain tissue was previously published^17^. Additionally, two hippocampal regions (CA1 and CA3) were independently evaluated using both OPEN-Stereo and the commercial system to assess cross-regional accuracy. Four mice were (re)evaluated in each brain region (Table 1). The percent congruence was determined using the following equation:

8 $$\begin{aligned} \% \text {C} = 100 \cdot \left( 1 - \frac{|N_{\text {commercial}} - N_{\text {OPEN Stereo}}|}{\left( N_{\text {commercial}} + N_{\text {OPEN Stereo}} \right) /2} \right) \end{aligned}$$

where C is the percent congruence between the estimate *N* number of cells from the two systems, commercial vs OPEN-Stereo.

### Calculating the estimated number of cells

The archetypal equation for estimating the number of cells in a volume was presented previously (Eq. 7). In the setup for OPEN-Stereo, the archetypal equation for estimating the number of cells in a volume can be written straightforward as (Eq. 9):

9 $$\begin{aligned} N = \sum _{n} (k^2 \cdot \delta s \cdot t/d) \end{aligned}$$

where *n* is the number of cells counted, *k* is the multiplier of the sampled area (Supplementary Fig. 3), $\delta s$ is the number of sections between counted sections, *t* is the measured tissue thickness, and *d* is the disector height. While *k*, $\delta s$, and *d* are user inputs, *t* is generated as part of running OPEN-Stereo.

### $\textbf{K}$-matrix: automated X-Y local scale calibration using a single feature point

As the first step of calibration, the user must choose a reference objective (e.g., 10×) to map the X-Y pixel coordinates to the X-Y stage motor coordinates. This process requires the user to set the objective to 10× (or the reference objective of choice) and focus on a region with at least one visually identifiable feature point. By pressing the [Calibrate] button (Supplementary Fig. 4a), the calibration sequence is initiated. The user clicks on a visual feature point on the screen, after which the automated sequence moves the X-Y stage by predetermined distances and tracks the feature point to extract the $\textbf{K}$ matrix for the reference scale, as outlined in Fig. 1a. This step must be completed first before creating the inter-objective calibration ($\textbf{L}$-matrix). A step-by-step calibration video is available at^15^.

### L-matrix: inter-objective calibration using a calibration grid at 10$\mu \textrm{m}$ Resolution

Begin by centering the view on the calibration grid at the reference objective (10×). Position the calibration grid so that the points selected at 10× are also visible at the target objective (100×) without repositioning the XY stage. Press the [Calib L] button to initiate the inter-objective calibration sequence (Supplementary Fig. 4b). The program will prompt for the objective choice and guide the user to refocus on the grid. The user selects four calibration grid points at the reference magnification (Supplementary Fig 4c). These four points will serve as the reference points for all subsequent inter-objective transformations. After the 4th point is selected, the program will prompt the user to change objectives (Supplementary Fig 4d). Once focused at the secondary target objective, the user must then reselect the same four reference points at the new magnification, 100× (Supplementary Fig. 4e). This step establishes a transformation between the intermediate reference scale and all other magnifications.

After the inter-objective (10× to 100×) calibration, the user can select points via the [Pick Point] button (Supplementary Fig. 4f) at both the 10× and 100× scales. The system then determines the global coordinates for all selected points, allowing consistent navigation across the 10× and 100× magnifications. Points can be plotted using the [Plot Points] button (Supplementary Fig. 4g). It is through this paradigm that the system establishes a transformation between the intermediate reference 10× scale and all other objective pairs, such as 4× or 100×. By combining the previously calculated 100×-to-10× and 10×-to-4× transformations, the system computes a composite transformation matrix for 4×-to-100×. Thus, calibration is only required between each objective level and the 10× reference level, rather than between every possible objective pair.

### Integration and creation of the OPEN-Stereo system

Permanent links for all necessary programs are provided on the OPEN-Stereo GitHub website (https://github.com/OPEN-stereology/OPEN-stereology_manuscript)^15^, and videos provide walk-through tutorials for how to 1) calibrate the stage and objectives and 2) perform a walk-through example case for counting cells in the hippocampal CA1 region.

### Calibration accuracy

Typical examples of calibration accuracy were determined using the 10× and 100× objectives. One point was selected at the center of a 4-square (Supplementary Fig. 5a, white cross). Plotting that point at 100× (Supplementary Fig. 5b, white cross), reveals a slightly shifted outline from the black grid. Given that each square of the calibration grid is 10$\mu \text {m}$, based on the shift of the outline we estimate the average tolerance to be within 2-3$\mu \textrm{m}$. After changing the objective to 10×, the same point was re-plotted (Supplementary Fig. 5c, white cross). Here the misalignment is negligible, < 1$\mu \textrm{m}$. Additional points were selected using the 10× objective (Fig. 5c, white circle and additional points in magenta, at the intersection of the grid lines). These points were then plotted using the 100× objective (Supplementary Fig. 5d, white circle). Here the misalignment appears to be 1-2$\mu \textrm{m}$ up and 1$\mu \textrm{m}$ to the right. These experiments confirm that the calibration fidelity is within the tolerance required for stereology.

### Validation

In order to validate our system we re-counted mouse cases that had been previously counted using a commercial system. The University of California at San Diego’s animal subjects committee approved all experiments, and all experiments were carried out in accordance with relevant guidelines and regulations. A total of 12 cases were used for validation using different brain regions and different antibodies (Table 1). Cases 1-8 were immunostained with pan-neuronal marker NeuN (1:500, Millipore). Cases 9-12 were immunostained with ChAT according to^17^.

## Data Availability

The repository for the OPEN-Stereo software, along with links to tutorials, is hosted on GitHub at https://github.com/OPEN-stereology/OPEN-stereology_manuscript.
